# Preventing Adolescents' Diabesity: Design, Development, and First Evaluation of “Gustavo in Gnam's Planet”

**DOI:** 10.1089/g4h.2014.0107

**Published:** 2015-10-01

**Authors:** Daniela Marchetti, Federica Fraticelli, Francesco Polcini, Roberto Lato, Basilio Pintaudi, Antonio Nicolucci, Mario Fulcheri, Angelika Mohn, Francesco Chiarelli, Giacoma Di Vieste, Ester Vitacolonna

**Affiliations:** ^1^ Department of Psychological Sciences, Humanities and the Territory, “G. d'Annunzio” University, Chieti-Pescara, Chieti, Italy.; ^2^ Department of Medicine and Aging, “G. d'Annunzio” University, Chieti-Pescara, Chieti, Italy.; ^3^ “G. d'Annunzio” University, Chieti-Pescara, Chieti, Italy.; ^4^ Department of Clinical Pharmacology and Epidemiology, Fondazione Mario Negri Sud, S. Maria Imbaro, Chieti, Italy.; ^5^ Center of Excellence on Aging, “G. d'Annunzio” University Foundation, Chieti-Pescara, Chieti, Italy.

## Abstract

***Objective:*** The goal of this study was to design, develop, and evaluate a game for health, “Gustavo in Gnam's Planet” (“Gustavo”), aimed to improve knowledge on healthy foods and to increase consumption of healthy foods.

***Subjects and Methods:*** Eighty-three high school students were enrolled in the study. The game was designed and developed by a multidisciplinary team. Behavioral change theories were adopted to guide the design of the health messages. Participants were assessed about food frequency, healthy food knowledge, and the game's interest.

***Results:*** Forty-seven subjects (mean age, 14.9±1.0 years; 72.3 percent males) completed the study. At posttest, participants showed significant higher scores (i.e., increased knowledge) in the questionnaire on knowledge of healthy foods (70.0±9.2 versus 71.3±10.0 for pretest and posttest, respectively; *P*<0.05). Improvements in healthy eating habits were also detected: higher frequency of consumption during a week of white meat (1 [1–2] versus 2 [1–2]; *P*=0.01), eggs (1 [1–1] versus 1 [1–2]; *P*=0.01], and legumes (1 [0–1] versus 1 [1–2]; *P*=0.03) and lower frequency of consumption of sugar-containing packaged snacks (1 [0–1] versus 0 [0–1]; *P*=0.009). Most of the participants found the game easy to use and clear in its content. Half of the participants found the game interesting.

***Conclusions:*** Our study shows that “Gustavo” is a promising tool for health education, in schools or in other environments. Limitations of the study and future directions are discussed.

## Introduction

Diabesity represents a new health epidemic; it consists of the coexistence of both type 2 diabetes mellitus (T2DM) and obesity.^1^ T2DM and obesity have great social and clinical relevance because of the number of affected subjects and the related serious complications. Recent estimates show an increasing and worrisome trend toward younger people developing both conditions. This situation has deep economic implications for all healthcare systems: If preventive measures are not undertaken, the costs will soon become unsustainable.^2^

T2DM is a largely preventable disease; in this respect, promoting a healthy lifestyle is important and can be effective in young people. As described in a recent review, studies among children and adolescents clearly point out that the largest part of these populations poorly adheres to the Mediterranean diet also in the Mediterranean region, showing an increase in the intake of processed foods and saturated fat.^3^ The Mediterranean diet is the traditional dietary pattern of Mediterranean countries and is characterized by a high intake of olive oil, fruits and nuts, vegetables, legumes, cereals, fish, and seafood and a low intake of dairy and meat products.

Despite notable efforts to halt the growing rates of youth overweight or obesity, to date, strong evidence to support favorable effects of obesity prevention programs for adolescents is still lacking.^4^ Adolescence is a crucial stage for individual development, and successful completion of this stage is highly related to health. There is a need to develop more appealing tools for adolescents to promote effective prevention.

Recent studies suggest that serious games are interesting and innovative tools useful to influence attitudes, beliefs, and behaviors,^5,6^ more than other forms of communication-based media, such as brochures and Web sites. Serious games use entertainment technology to teach, train, or change the behavior, encouraging active engagement and processing of information from the users.^7–9^

Games for health (G4Hs) (or “health games”) are games with a focus on health care and physical and mental fitness.^6,10,11^ Popular application areas of health games are nutrition, physical training, education, and prevention.^12,13^ The game-based learning principles target intrinsic motivation, learning through fun, authenticity, self-reliance/autonomy, and experiential learning. The mechanisms adopted include rules, clear but challenging goals, fantasy, progressive levels of difficulty, interactivity, player control, uncertainty, feedback, and a social element.^14,15^

G4Hs can be divided into sedentary and active videogames: The first type uses a game controller, a keyboard, or a mouse, and the second is controlled with the motion of the player. Parisod et al.^16^ recently published a review on the effectiveness of digital games in children's health promotion. Active videogames seem to be effective in increasing energy expenditure and promoting physical activity. On the other hand, the evidence supporting the effectiveness of sedentary games is promising, but a large gap in knowledge was found related to this area. In fact, only a few sedentary health games are available because they require many resources for their developmental process.^16^

International studies focusing on nutrition digital games evaluation found positive results. Peng^17^ evaluated a nutrition education and weight management computer game. The study showed that the game was successful in delivering nutrition information and changing psychosocial determinants of healthy eating in young adults with an average age of 20 years. A project targeting younger participants (children 10–12 years old) was developed by Baranowski et al.^18^ They delivered noncommercial and online computer games designed to work as a set to address diet, physical activity, and energy balance. This randomized clinical trial study showed that children playing these computer games increased their fruits and vegetables consumption. Similarly, in a more recent study,^19^ an online health videogame for improving healthy diet and physical activity in primary school students (10–11-year-old children) was evaluated. The study documented a significant increase in positive attitudes toward healthy eating and healthy eating self-efficacy, and a high acceptability of the videogame was reported by the participants. Nevertheless, none of these studies evaluated the effectiveness of a nutrition education game on a target group of high school students (adolescents 14–18 years of age).

The aim of this study was to design, develop, and evaluate a sedentary G4H called “Gustavo in Gnam's Planet” (hereinafter called “Gustavo”), targeting adolescents between 14 and 18 years of age. To our knowledge this is the first study in Italy aimed at testing a G4H for prevention programs in youth.

After describing the design and development process, we present a pilot intervention study testing if playing “Gustavo” could improve knowledge on and enhance consumption of healthy foods in adolescents attending high school.

## Materials and Methods

### Game design and theoretical framework

“Gustavo” is an original Web game developed during a project of health promotion by a multidisciplinary team. The multidisciplinary team, as suggested by Baranowski et al.,^15^ included both “fun-ness” professionals, like a sound professional, a computer programmer, an artist, and a writer, and “serious-ness” professionals, like an expert in nutrition and metabolic diseases, a psychologist, and a dietitian. The model of the game has been defined from a technical, medical, nutritional, and psychological point of view according to recent specialized literature.^15,20^

To increase the likelihood of success of our G4H, we used a theoretical framework. Psychology theories attesting specific factors involved in behavior change are the Transtheoretical Model of Change, the Social Cognitive Theory, the Self-Determination Theory, and the Elaboration Likelihood Model. The central organizing construct of the Transtheoretical Model of Change^21^ is the Stages of Change, in which behavior change is a process involving progress through a series of five stages (from Precontemplation to Maintenance). The Processes of Change are eased by three factors of behavioral change: self-efficacy, locus of control, and decisional balance. Self-efficacy is also the core concept of the Social Cognitive Theory.^22^ Social Cognitive Theory suggests that behavior, personal factors, and the environment are interrelated and work together to achieve behavior change. Specifically, personal factors involved are behavior-specific knowledge, self-efficacy, and/or self-regulatory skills development. According to Self-Determination Theory,^23^ one additional factor tied to behavior change is intrinsic motivation. Moreover, the Elaboration Likelihood Model^24^ proposes that gaining and keeping a person's attention is the first step in getting a person to process the information in a message to promote behavior change.

An integrative approach, aimed at achieving a high efficacy of “Gustavo” to change knowledge and behavior, was used. Some of the effective constructs of behavioral change theories described above were endorsed to design and develop the Web game and its health messages, such as attention retention, self-efficacy, self-regulatory skill development, and intrinsic motivation. The Web game was developed to create an entertaining and engaging environment within which behavior-specific knowledge improvement and self-efficacy increase can occur. This was developed through modeling guided by self-identification with the game characters, feedback, and rewards provided both during the stages and at the end of any stage (i.e., positive feedback and rewarding points are provided to players when they chose healthy foods). Self-regulatory skill acquisition and confidence in doing the new behavior are promoted through the elaboration of the healthy messages provided during the game and the direct experience of the effects of personal choices in the simulated environment (leading to self-efficacy).

As described in many articles,^7,15,17,20,25^ one of the key features of games is fun. The teamwork was attentive to put in the game elements of fun (e.g., obstacles, unexpected events, music, interaction between characters), thereby maintaining participants' attention and motivation through the fun experience. Finally, to ensure participants' engagement the teamwork developed a story of the main character and his friend (Table 1).

**Table 1.** Characteristics of “Gustavo in Gnam's Planet”

*Characteristic*	
Health topic(s)	Diabesity prevention
Targeted age groups	14–18 years old
Other targeted group characteristics	Italian speaking language
Short description of game idea	To learn Mediterranean diet and behavioral moderation by playing
Target player(s)	Individual
Guiding knowledge or behavior change theory(ies), models, or conceptual framework(s)	Transtheoretical Model of Change, Social Cognitive Theory, Self-Determination Theory, Elaboration Likelihood Model
Intended health behavior changes	Improvement of consumption of healthy foods and reduction of junk/unhealthy foods
Knowledge element(s) to be learned	Mediterranean diet
Behavior change procedure(s) (taken from the Michie inventory) or therapeutic procedure(s) used	Goal Feedback Reward Punishment Shaping Instructions
Clinical or parental support needed?	No
Data shared with parent or clinician	Yes, data are shared with parents and teachers.
Type of game	Endless running, educational
Story	
Synopsis (including story arc)	Gustavo is the game's young hero, a cheerful kid who lives an incredible adventure with his faithful cat LOL. Gustavo and LOL are inseparable friends, sharing a big love for runs in the open air, jumps, and a healthy lifestyle. One night, LOL wakes Gustavo up because he is hungry. Still half asleep, Gustavo takes the cat to the kitchen to get a glass of milk from the fridge. But, because of a manufacturing fault, the fridge transforms itself into an interdimensional portal, through which Gustavo is transported faraway to the planet GNAM, an unknown world…with plenty of food! Only LOL knows the secret of planet GNAM: The only way for Gustavo to go back home is to adopt a healthy lifestyle. Nothing is easier for Gustavo, who loves running, jumping, and eating healthy food! Will he be able to get back home safe, sound, and on time for breakfast? Only you can help him!
How the story relates to targeted behavior change	While playing the game, subjects acquire specific information, and the bonus/penalty mechanism in the three levels acts as a stimulus to transform the choices they make in the game into practical deeds in their real life (i.e., choosing healthy food and avoiding junk food).
Game components	
Player's game goal/objective(s)	To reach the end level with the highest score while helping Gustavo to maintain a healthy lifestyle
Rules	To finish the game the subject has to successfully complete three levels. To complete each level a player can make up to three penalties and has to dodge obstacles. Penalties are given when the game's protagonist eats unhealthy foods, which are shown before starting to play and are also highlighted while playing.Obstacles concern the game's physics and setting's morphology (e.g., do not fall down into cliffs, do not bump into rock walls, and so on). The score is always the same: 100 points for each bonus food eaten. The gained score is shown at the end of each level, and the total score is shown at the end of the third and final level.
Game mechanic(s)	The game uses “endless running” mechanics: The protagonist runs at a fixed speed. The user's only possible interaction is the jump. The jump, which is performed by pressing the space bar, allows Gustavo to eat or avoid foods, dodge obstacles, and choose a path.
Procedures to generalize or transfer what's learned in the game to outside the game	Some foods allow Gustavo to maintain a healthy lifestyle and earn scores, whereas an excessive consumption of unhealthy foods leads to starting the level once again.
Virtual environment	
Setting (describe)	There are no user-customizable settings. The game's physics are fixed and set up during the development stage.
Avatar	
Characteristics	Only one avatar is available: Gustavo. It represents a child created by using a mixed two-dimensional illustration technique.
Abilities	Apart from his ability to jump up to five times his height, the avatar has no other abilities or powers.
Game platform(s) needed to play the game	The game has been developed for the PC platform only. To play, users need a computer (with Windows, Macintosh, or Linux operating system) connected to the Internet. In addition, it is necessary to install a browser and the free “Unity” plug-in.
Sensors used	No sensors used
Estimated play time	It depends on the player's expertise: A beginner player might take at least 2 hours to finish the game, whereas an expert player can finish it in 30 minutes.

### Game structure and rules

**FIG. 1.**
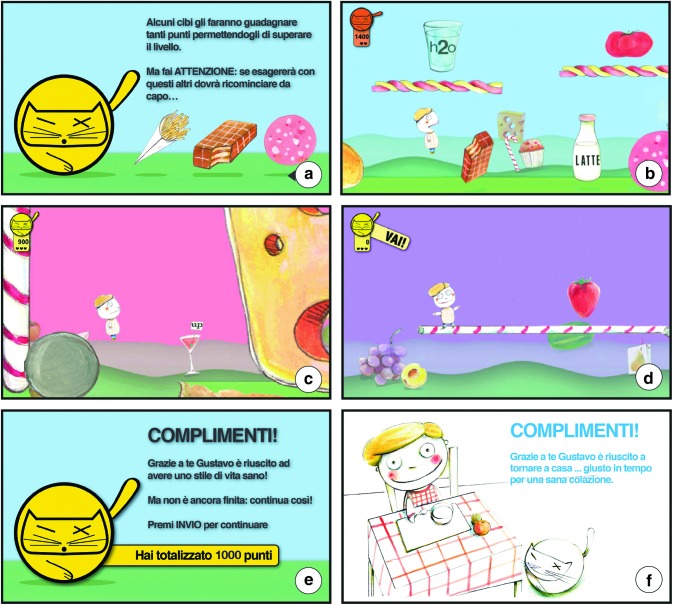
Screenshots of “Gustave in Gnam's Planet.” **(a)** Splash screen with the textual and visual instructions to overcome the level. The penalties of the first level are displayed. **(b)** The first level. **(c)** The second level. **(d)** The beginning of the third level. **(e)** End level splash screen. A positive feedback and the invitation to continue playing the game are displayed. **(f)** End game splash screen with the reward message: “Congratulations! Gustavo successfully came back home with your help.. just in time for a healthy breakfast.” (Color images available online at www.liebertonline.com/g4h)

“Gustavo” combines a classic two-dimensional videogame platform structure with the mechanical characteristics of the endless runner genre (Fig. 1). The protagonist of the game has to move between various obstacles, its movement is automatic, and the player has to use only a key to enable him to jump. The game provides a mechanism of bonuses and penalties: some foods allow the Gustavo protagonist to maintain a healthy lifestyle and to gain points (e.g., vegetables, legumes, white meat, fish, olive oil), but the player will have to help Gustavo not to overdo with unhealthy foods (e.g., chips, sugar-sweetened beverages, hot dog, mayonnaise). Indeed, when Gustavo eats three unhealthy foods in the same level, the player has to restart the level from the beginning. This methodology allows the application of the important concept of “moderation.”

Playing “Gustavo” does not require any special skills or previous experience in videogames, but thanks to the use of a curve of increasing difficulty among the three levels of the game, players are encouraged to have fun up to the end of the game. The player is encouraged to play several times to discover all the secrets and to cover the different roads leading to the end of the three levels of playing.

### Participants and procedures

Participants were 83 healthy adolescents, 14–18 years of age (mean age, 16.1±1.3 years; 70.0 percent males), attending high school from two schools in Pescara, Italy. After the informed consent was orally obtained from students and their parents, students attended a meeting at school with an expert in nutrition and metabolic diseases. During the meeting the G4H “Gustavo” was presented, and the steps of the research were explained. Students were provided with personal credentials to access on a voluntary basis the reserved Web area, where they could find all the contents of the research.

The questionnaires for pretest and posttest evaluations were developed and delivered through LimeSurvey, an open source tool for online surveys. The Web game “Gustavo” was developed on the Unity platform. The reserved Web area displayed the instructions of the research, the links to LimeSurvey's pages for the two times of assessment, and the link to the Unity page of the Web game “Gustavo.”

All the participants were first required to complete pretest questionnaires and then to play the Web game for 1 week, at least half an hour per day; finally, they were asked to complete the posttest questionnaires. Participants were also informed that all the research activities had to be performed at home. All subjects (*n*=83) have agreed and have logged into the reserved area at least once; 47 had completed information on pretest and posttest questionnaires, and those were used for analyses.

### Measures

Basic demographic data (age and gender) were collected.

The healthy food knowledge questionnaire was specifically developed for this study to evaluate the knowledge of participants about healthy foods. This self-administered tool has been developed by the dietitian and the psychologist on the multidisciplinary team considering the Mediterranean diet principles and the psychometric principles. After its development, each member of the multidisciplinary team approved the questionnaire. For 22 kinds of food that players run into in the game, participants were asked to indicate on a 5-point Likert-type scale ranging from “disagree” (0) to “agree” (4) the level they believe each food should be recommended in a healthy diet. The scale provides a total score, ranging from 0 to 88, with a higher score indicating greater knowledge about healthy foods. Cronbach's alpha coefficients for pretest and posttest showed a good reliability of the measure (Table 2).

**Table 2.** Results of Pretest and Posttest of Healthy Food Knowledge

	*Mean±SD*	*Alpha*	P^a^
Pretest	70.0±9.2	0.81	0.02
Posttest	71.3±10.0	0.84	

Data were collected in April 2013 in Pescara, Italy

^a^ A *P* value<0.05 was considered for statistical significance.

SD, standard deviation.

A food frequency questionnaire was developed specifically for the study, to assess the frequency of consumption of common foods in the previous week. The starting point in the development of the measure was a survey used by the Italian National Institute of Health to assess the lifestyle of young people. The questionnaire was modified—according to the needs of the study—increasing the response options and the number of foods. Participants were asked to mark on a 5-point Likert-type scale their consumption in the previous week of 33 foods. Scores for each food could range from 0 (none of the days of the week) to 4 (every day of the week). Each single food score was used for data analysis.

The Interest questionnaire consisted of eight items investigating the acceptability and opinions about the game's characteristics such as structural features (i.e., graphic and sound), enjoyment and playability of the game, relevance, and self-perception about learning through the game. Examples of items are “I enjoyed playing this game,” “Overall, I believe the game is interesting,” and “The game is easy to play.” The respondents answered on a 5-point Likert-type scale ranging from disagree (0) to agree (4).

### Statistical analysis

Data were collected for pretest and posttest in 2013. Anonymity and confidentiality of participants were secured by personal credentials. Data were summarized as mean±standard deviation for continuous variables and frequency (percentages) for categorical variables. Food frequency is reported as median and range. The primary efficacy variable was absolute change in knowledge on healthy diet score. Pretest posttest levels of knowledge on healthy diet and frequency consumption of foods were compared using the Wilcoxon signed-rank sum test. Statistical analysis was carried out with SPSS statistical package version 17.0 software (SPSS, Inc., Chicago, IL).

## Results

Forty-seven participants (mean age, 14.9±1.0 years; 72.3 percent males) had complete information available. Subjects significantly improved their knowledge on healthy diet (Table 2) after playing the game (*P*=0.02).

Comparisons of foods frequency consumption revealed that participants showed significant higher frequency consumption of white meat (*P*=0.01), eggs (*P*=0.01), and legumes (*P*=0.03) and lower consumption of sugar-containing packaged snacks (*P*=0.009) after having played “Gustavo” for 1 week (Table 3). Fish consumption increased from baseline to the end of the study, even if statistical significance was not reached (*P*=0.05).

**Table 3.** Comparison of Food Frequency Consumption Before and After Playing the Game

*Food*	*Pretest*	*Posttest*	P^a^
Pasta, rice, other cereals	3 (2–4)	4 (2–4)	0.16
Bread	3 (2–4)	3 (2–4)	0.46
Potato	1 (1–1)	1 (1–1)	0.97
Milk, yogurt	4 (1–4)	3 (1–4)	0.65
Croissant, brioches	0 (0–1)	1 (0–1)	0.38
Sugar-containing packaged snacks	1 (0–1)	0 (0–1)	0.009^b^
Homemade cake	1 (0–2)	1 (0–2)	0.29
Biscuit	1 (1–3)	1 (1–3)	0.57
Spreadable cream	0 (0–1)	0 (0–1)	0.49
Breakfast cereals	1 (0–3)	1 (0–3)	0.95
Crackers, salty snack	0 (0–2)	1 (0–2)	0.30
Chips	0 (0–1)	0 (0–1)	0.13
Pizza	1 (1–1)	1 (1–2)	0.53
Hamburger, hot dog	0 (0–1)	0 (0–1)	0.21
Sandwich, toast	1 (1–2)	1 (0–2)	0.33
Red meat	2 (1–2)	2 (1–2)	0.07
White meat	1 (1–2)	2 (1–2)	0.01^b^
Fish	1 (0–1)	1 (0–2)	0.05
Fresh cheese	1 (0–1)	1 (1–2)	0.17
Cheese	1 (0–2)	1 (0–2)	0.12
Eggs	1 (1–1)	1 (1–2)	0.01^b^
Cold cuts	2 (1–2)	2 (1–2)	0.79
Legumes	1 (0–1)	1 (1–2)	0.03^b^
Vegetables	2 (1–3)	2 (1–3)	0.31
Fresh fruit	3 (2–4)	3 (2–4)	0.09
Dried fruit	0 (0–0)	0 (0–1)	0.13
Fruit juice	1 (0–3)	1 (0–2)	0.15
Soft drink	0 (0–2)	1 (0–1)	0.81
Slice of cake, dessert	1 (0–2)	1 (0–2)	0.62
Extra virgin olive oil	3 (2–4)	3 (2–4)	0.14
Sauces (mayonnaise, ketchup)	0 (0–1)	0 (0–1)	0.41
Butter, margarine, heavy cream	0 (0–1)	0 (0–1)	0.59
Precooked food	0 (0–0)	0 (0–0)	0.41

Data were collected in April 2013 in Pescara, Italy. Data are expressed as median (interquartile range) of the participants' scores. A score of 0 means none of the days of the last week, 1 means 1 or 2 days of the last week, 2 means 3 or 4 days of the last week, 3 means 5 or 6 days of the last week, and 4 means every day of the last week.

^a^ A *P* value<0.05 was considered for statistical significance. Comparisons were calculated by using the Wilcoxon signed-rank sum test.

^b^ Indicates statistical significance.

When considering the interest questionnaire, 76.6 percent of subjects judged “Gustavo” easy to use, and 87.3 percent of participants declared it was clear in its contents. Half of the participants found the game interesting. Only relatively few of the subjects (17.1 percent) recognized having acquired new skills, at variance with what emerged from the results of the questionnaire on knowledge.

## Discussion

Children and adolescents are the ideal target population for the prevention of metabolic diseases. Because videogaming is a common pastime among adolescents, videogame-based interventions may constitute one component of a broader integrative approach for preventive health interventions.

A multidisciplinary team designed, developed, and first evaluated a computer Web game aimed to prevent diabesity among Italian adolescents. The pilot intervention study has two main practical results. First, it showed that playing a game like “Gustavo” can be useful to increase awareness on healthy food. As an example, participants could ignore the benefits of eating legumes or white meat because of lack of knowledge of their parents or cultural reasons. In this way, “Gustavo” represents a didactic and informative game. Second, and more important, is that playing the game resulted in a measurable change. Participants increased the consumption of healthy foods and decreased that of unhealthy foods. This consistency of actions reflects the effect of a more or less conscious choice with important consequences in terms of gaining health and preventing metabolic diseases.

Because participants played the Web game for 1 week only, our results are encouraging: The use of “Gustavo” for extended periods can be an enjoyable and promising tool to use for health prevention programs, in schools or in other environments. The first evaluation of our GH4 showed that playing for 1 week is not enough to fully change the nutritional habits of adolescents. However, 1 week is a suitable interval of time to increase knowledge about healthy diet according to the nutritional notions of the Mediterranean diet. Another interesting result is that participants have acquired specific nutrition information through “Gustavo,” even if most of them didn't recognize it consciously. We can consider this result as a manifestation, typical of serious games, of the nonperception of learning when instead doing it.^26,27^

The results are consistent with previous studies that showed that a behaviorally targeted videogame can produce significant improvements in nutritional knowledge and dietary habits.^11,17,28^ Furthermore, the study evaluated a new G4H with some distinctiveness from previous nutrition education games: “Gustavo in Gnam's Planet” is a Web game; it uses the endless running mechanics; it is targeted at adolescents; and it was developed by a multidisciplinary team using an integrative approach of behavior change theories. One of the most serious problems of prevention programs is their sustainability.^4^ Our G4H is reusable, the program delivery is standardized, and the burden on the system can be reduced.

The main limits of our study include the short time of intervention, the small sample size, the lack of a control group, the lack of a follow-up measure, and the impossibility to keep track of the time that participants spent in the game. Despite these notable methodological limitations, the findings of our pilot intervention study could be used to inform a more stringent study that would establish greater generalizability. Another possible limitation is that participants played and filled in pretest and posttest questionnaires at home. As argued by Baranowski et al.,^15^ a game offered at home may be less attractive than the same game played at school as an alternative to a didactic classroom lesson. This may explain the rate of dropouts during the study and of the interest for the game expressed by only half of the participants. It will be important to evaluate the acceptability of our G4H when it is played at school instead at home, and we plan to do so in future studies.

## Conclusions

A G4H intervention to prevent diabesity may be particularly effective in childhood and adolescence. Overall, it seems that “Gustavo” can increase nutrition knowledge and partially change dietary behavior of adolescents. Although the findings of this study are quite positive, longer experiments with larger samples need to be conducted in the future to further examine the effectiveness of the G4H approach for nutrition education. We intend to repeat this intervention with a control group, and we plan to follow up adolescents to evaluate the long-term effect of a school-based diabesity prevention program by using our G4H.

## References

[1] Galli-Tsinopoulou A, Grammatikopoulou MG, Stylianou C, et al. Diabese youngsters have 3.7 more chances in developing metabolic syndrome compared with the obese. J Endocrinol Invest 2010; 33:549–553 20190555 10.1007/BF03346646

[2] International Diabetes Federation. IDF Diabetes Atlas, 6th ed. Brussels: International Diabetes Federation; 2013 www.idf.org/diabetesatlas (accessed 13, 2014)

[3] Naska A, Trichopoulou A Back to the future: The Mediterranean diet paradigm. Nutr Metab Cardiovasc Dis 2014; 24:216–219 24462051 10.1016/j.numecd.2013.11.007

[4] Waters E, de Silva-Sanigorski A, Hall BJ, et al. Interventions for preventing obesity in children. Cochrane Database Syst Rev 2011; (12):CD001871 22161367 10.1002/14651858.CD001871.pub3

[5] Becker K The magic bullet. A tool for assessing and evaluating learning potential in games. Int J Game Base Learn 2011; 1:19–31

[6] Papastergiou M Exploring the potential of computer and video games for health and physical education: A literature review. Comput Educ 2009; 53:603–622

[7] Baranowski T, Buday R, Thompson DI, et al. Playing for real: Video games and stories for health-related behavior change. Am J Prev Med 2008; 34:74–82 18083454 10.1016/j.amepre.2007.09.027 PMC2189579

[8] Connolly TM, Boyle EA, MacArthur E, et al. A systematic literature review of empirical evidence on computer games and serious games. Comput Educ 2012; 59:661–686

[9] Ma M Introduction to serious games development and applications. Entertain Comput 2011; 2:59–60

[10] Brox E, Fernandez-Luque L, Tøllefsen T Healthy gaming—Video game design to promote health. Appl Clin Inform 2011; 2:128–142 23616865 10.4338/ACI-2010-10-R-0060 PMC3631924

[11] Johnson-Glenberg MC, Hekler EB “Alien Health Game”: An embodied exergame to instruct in nutrition and MyPlate. Games Health J 2013; 2:354–361 26197077 10.1089/g4h.2013.0057

[12] Fuchslocher A, Niesenhaus J, Krämer N Serious games for health: An empirical study of the game “Balance” for teenagers with diabetes mellitus. Entertain Comput 2011; 2:97–101

[13] Susi T, Johannesson M, Backlund P Serious Games—An Overview. Technical Report HS-IKI-TR-07-001. Skövde, Sweden: School of Humanities and Informatics, University of Skövde; 2007 http://his.diva-portal.org/smash/get/diva2:2416/FULLTEXT01 (accessed 718, 2013)

[14] Perrotta C, Featherstone G, Aston H, et al. Game-Based Learning: Latest Evidence and Future Directions. 2013 www.nfer.ac.uk/publications/GAME01/GAME01.pdf (accessed 92, 2014)

[15] Baranowski T, Buday R, Thompson D, et al. Developing games for health behavior change: Getting started. Games Health J 2013; 2:183–190 24443708 10.1089/g4h.2013.0048 PMC3892986

[16] Parisod H, Pakarinen A, Kauhanen L, et al. Promoting children's health with digital games: A review of reviews. Games Health J 2014; 3:145–156 26196173 10.1089/g4h.2013.0086

[17] Peng W Design and evaluation of a computer game to promote a healthy diet for young adults. Health Commun 2009; 24:115–127 19280455 10.1080/10410230802676490

[18] Baranowski T, Baranowski J, Thompson D, et al. Video game play, child diet, and physical activity behavior change: A randomized clinical trial. Am J Prev Med 2011; 40:33–38 21146765 10.1016/j.amepre.2010.09.029 PMC3032382

[19] Schneider KL, Ferrara J, Lance B, et al. Acceptability of an online health videogame to improve diet and physical activity in elementary school students: “Fitter Critters.” Games Health J 2012; 1:262–268 24761317 10.1089/g4h.2012.0009 PMC3833367

[20] Baranowski T, Lieberman D, Buday R, et al. Videogame mechanics in games for health. Games Health J 2013; 2:194–204 26192223 10.1089/g4h.2013.0617

[21] Prochaska JO, DiClemente CC Stages and processes of self-change of smoking: Toward an integrative model of change. J Consult Clin Psychol 1983; 51:390–395 6863699 10.1037//0022-006x.51.3.390

[22] Bandura A Social Foundations of Thought and Action: A Social Cognitive Theory. Englewood Cliffs, NJ: Prentice-Hall; 1986

[23] Ryan RM, Deci EL Self-determination theory and the facilitation of intrinsic motivation, social development, and well-being. Am Psychol 2000; 55:68–78 11392867 10.1037//0003-066x.55.1.68

[24] Petty RE, Cacioppo JT Communication and Persuasion. Central and Peripheral Routes to Attitude Change. New York: Springer-Verlag; 1986

[25] Marsh T Serious games continuum: Between games for purpose and experiential environments for purpose. Entertain Comput 2011; 2:61–68

[26] Rosas R, Nussbaum M, Cumsille P, et al. Beyond Nintendo: Design and assessment of educational video games for first and second grade students. Comput Educ 2003; 40:71–94

[27] Wenglinsky H Does It Compute? The Relationship Between Educational Technology and Student Achievement in Mathematics. Princeton, NJ: Educational Testing Service; 1998

[28] Turnin MC, Tauber MT, Couvaras O, et al. Evaluation of microcomputer nutritional teaching games in 1,876 children at school. Diabetes Metab 2001; 27:459–464 11547219

